# Di-μ-hydroxido-κ^4^ *O*:*O*-di-μ-perchlorato-κ^4^ *O*:*O*′-bis­[(2,2′-bi­pyridine-κ^2^ *N*,*N*′)copper(II)]

**DOI:** 10.1107/S1600536813027852

**Published:** 2013-10-16

**Authors:** B. Saravanan, A. Jayamani, N. Sengottuvelan, G. Chakkaravarthi, V. Manivannan

**Affiliations:** a Centre for Research and Development, PRIST University, Vallam, Thanjavur 613 403, India; b Department of Chemistry, DDE, Alagappa University, Karaikudi 630 003, India; c Department of Physics, CPCL Polytechnic College, Chennai 600 068, India

## Abstract

In the title binuclear copper(II) complex, [Cu_2_(ClO_4_)_2_(OH)_2_(C_10_H_8_N_2_)_2_], the Cu^II^ ion is coordinated in the form of a Jahn–Teller distorted octahedron by two bi­pyridine N atoms, two perchlorate O atoms and two hydroxide O atoms, and displays a distorted octa­hedral geometry. The mol­ecule belongs to the symmetry point group *C*_2*h*_. The Cu^II^ ion is located on a twofold rotation axis and the hydroxide and perchlorate ligands are located on a mirror plane. Within the dinuclear mol­ecule, the Cu⋯Cu separation is 2.8614 (7) Å. The crystal structure exhibits O—H⋯O, C—H⋯O and π–π [centroid–centroid distance = 3.5374 (13) Å] inter­actions.

## Related literature

For the biological activity of copper complexes, see: Müller *et al.* (2003 ▶); Lo *et al.* (2000 ▶). For related strucutures, see: Li *et al.* (2009 ▶); Shaikh *et al.* (2012 ▶); Wang *et al.* (2010 ▶).
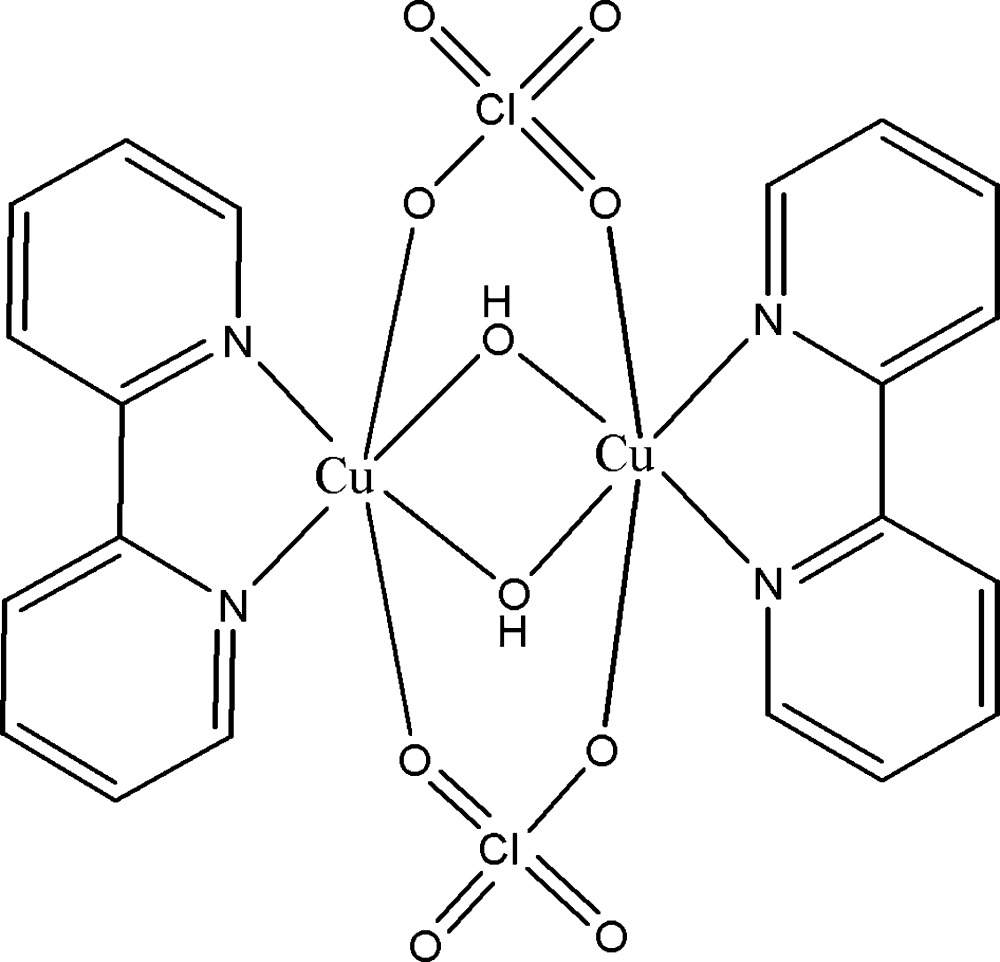


## Experimental

### 

#### Crystal data

[Cu_2_(ClO_4_)_2_(OH)_2_(C_10_H_8_N_2_)_2_]
*M* *_r_* = 672.36
Monoclinic, 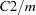

*a* = 13.6014 (12) Å
*b* = 15.2064 (13) Å
*c* = 6.2738 (6) Å
β = 113.587 (3)°
*V* = 1189.19 (19) Å^3^
*Z* = 2
Mo *K*α radiation
μ = 2.08 mm^−1^
*T* = 295 K
0.24 × 0.20 × 0.18 mm

#### Data collection

Bruker Kappa APEXII diffractometer
Absorption correction: multi-scan (*SADABS*; Sheldrick, 1996 ▶) *T*_min_ = 0.635, *T*_max_ = 0.706
4520 measured reflections
1516 independent reflections
1330 reflections with *I* > 2σ(*I*)
*R*_int_ = 0.022

#### Refinement

*R*[*F* ^2^ > 2σ(*F* ^2^)] = 0.026
*wR*(*F* ^2^) = 0.077
*S* = 1.03
1516 reflections
95 parameters
1 restraint
H atoms treated by a mixture of independent and constrained refinement
Δρ_max_ = 0.44 e Å^−3^
Δρ_min_ = −0.31 e Å^−3^

### 

Data collection: *APEX2* (Bruker, 2004 ▶); cell refinement: *SAINT* (Bruker, 2004 ▶); data reduction: *SAINT*; program(s) used to solve structure: *SHELXS97* (Sheldrick, 2008 ▶); program(s) used to refine structure: *SHELXL97* (Sheldrick, 2008 ▶); molecular graphics: *PLATON* (Spek, 2009 ▶); software used to prepare material for publication: *SHELXL97*.

## Supplementary Material

Crystal structure: contains datablock(s) global, I. DOI: 10.1107/S1600536813027852/bt6938sup1.cif

Structure factors: contains datablock(s) I. DOI: 10.1107/S1600536813027852/bt6938Isup2.hkl

Additional supplementary materials:  crystallographic information; 3D view; checkCIF report

## Figures and Tables

**Table 1:** Hydrogen-bond geometry (Å, °)

*D*—H⋯*A*	*D*—H	H⋯*A*	*D*⋯*A*	*D*—H⋯*A*
O1—H1⋯O2^i^	0.81 (2)	2.34 (1)	3.134 (3)	169 (4)
C5—H5⋯O2^i^	0.93	2.52	3.381 (3)	153

Symmetry code: (i) 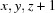.

**Fig. 1.**
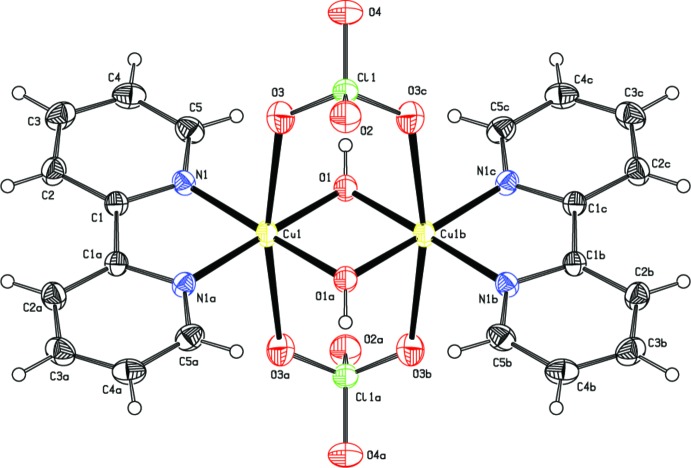
The molecular structure of the title compound, with atom labels and 30% probability displacement ellipsoids for non-H atoms. Symmetry codes : (a) -2-x, y, -1-z; (b) -2-x, -y, -1-z; (c) x, -y, z.

**Fig. 2.**
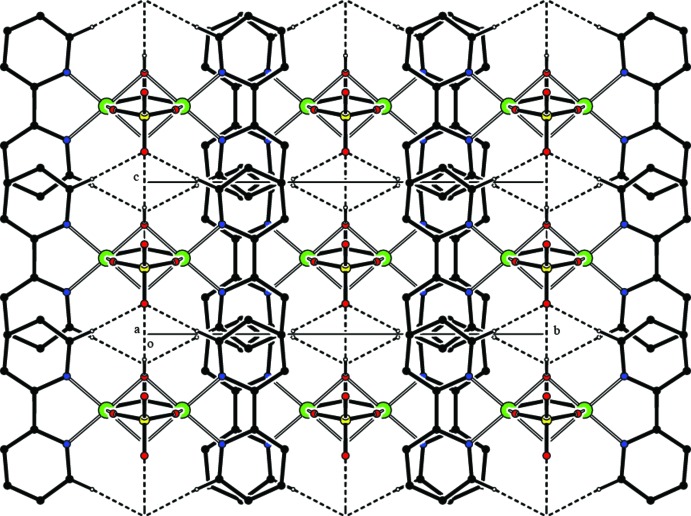
The packing of the title compound, viewed down a axis. Hydrogen bonds are shown as dashed lines. H atoms not involved in hydrogen bonding have been omitted.

## supplementary crystallographic information

### 1. Comment

Copper complexes have received much attention because of their interesting interactions with biological ligands to generate stable mixed coordinated complexes, which play a key role in life processes such as enzymatic catalysis, storage and conveyance of the matter, transfer of copper ions (Müller *et al.*, 2003; Lo *et al.*, 2000). In the molecular structure of the title compound (Fig. 1), the bond distances Cu1—N1 = 1.9865 (16) Å and Cu1—O1 = 1.9097 (13) Å agree with the reported similar structures (Shaikh *et al.*, 2012; Wang *et al.*, 2010). Each Cu^(II)^ cation is hexa-coordinated with two N atoms of bipyridine, two hydroxyl group O atoms bridging the copper cations and two O atoms of perchlorate anions, showing distorted octahedral environment (Fig. 1). The molecule belongs to the symmetry point group *C_2h_*. The two copper anions are separated by a distance of 2.8614 (7) Å, indicating a strong Cu^II^···Cu^II^ interaction which is comparable with the Cu^II^···Cu^II^ distance in the reported structure (Li *et al.*, 2009).

The crystal structure is stabilized by O—H···O, C—H···O (Fig. 2 & Table 1) and π–π [Cg1···Cg1^i^ distance = 3.5374 (13) Å; (i) -2-*x*, *y*, -*z*; Cg1 is the centroid of the ring (N1/C1/-C5)] interactions.

### 2. Experimental

To a solution of 2,2'-bipyridine (0.25 g, 1.60 mM) in 10 mL methanol, Cu(ClO_4_)_2_. 6H_2_O (0.59 g, 1.60 mM) in 10 mL of methanol, was slowly added dropwise with constant stirring. The mixture was stirred well at room temperature for about 3 h, the formed blue solution was then concentrated to one third of its volume, washed well (with water, methanol and ether) and dried under vacuum. The complex was then recrystallized in ethanol by the slow evaporation method to obtain X-ray quality single crystals of the complex, which appeared gradually after several days.

### 3. Refinement

The H atom of the hydroxyl O atom was located in a difference Fourier map and refined with the O1—H1 distance restrained to 0.82 (1)Å. All other H atoms were positioned geometrically and refined using riding model, with C—H = 0.93 Å and U_iso_(H) = 1.2U_eq_(C).

One reflection (1 1 0) was omitted from the final cycles of refinement owing to poor agreement.

### Crystal data

**Table:**

[Cu_2_(ClO_4_)_2_(OH)_2_(C_10_H_8_N_2_)_2_]	*F*(000) = 676
*M**_r_* = 672.36	*D*_x_ = 1.878 Mg m^−^^3^
Monoclinic, *C*2/*m*	Mo *K*α radiation, λ = 0.71073 Å
Hall symbol: -C 2y	Cell parameters from 4012 reflections
*a* = 13.6014 (12) Å	θ = 3.2–28.3°
*b* = 15.2064 (13) Å	µ = 2.08 mm^−^^1^
*c* = 6.2738 (6) Å	*T* = 295 K
β = 113.587 (3)°	Block, colourless
*V* = 1189.19 (19) Å^3^	0.24 × 0.20 × 0.18 mm
*Z* = 2	

### Data collection

**Table:**

Bruker Kappa APEXII diffractometer	1516 independent reflections
Radiation source: fine-focus sealed tube	1330 reflections with *I* > 2σ(*I*)
Graphite monochromator	*R*_int_ = 0.022
ω and φ scans	θ_max_ = 28.3°, θ_min_ = 3.3°
Absorption correction: multi-scan (*SADABS*; Sheldrick, 1996)	*h* = −17→17
*T*_min_ = 0.635, *T*_max_ = 0.706	*k* = −20→18
4520 measured reflections	*l* = −8→8

### Refinement

**Table:**

Refinement on *F*^2^	Primary atom site location: structure-invariant direct methods
Least-squares matrix: full	Secondary atom site location: difference Fourier map
*R*[*F*^2^ > 2σ(*F*^2^)] = 0.026	Hydrogen site location: inferred from neighbouring sites
*wR*(*F*^2^) = 0.077	H atoms treated by a mixture of independent and constrained refinement
*S* = 1.03	*w* = 1/[σ^2^(*F*_o_^2^) + (0.0419*P*)^2^ + 0.8702*P*] where *P* = (*F*_o_^2^ + 2*F*_c_^2^)/3
1516 reflections	(Δ/σ)_max_ < 0.001
95 parameters	Δρ_max_ = 0.44 e Å^−^^3^
1 restraint	Δρ_min_ = −0.31 e Å^−^^3^

### Special details

**Table:**

Geometry. All esds (except the esd in the dihedral angle between two l.s. planes) are estimated using the full covariance matrix. The cell esds are taken into account individually in the estimation of esds in distances, angles and torsion angles; correlations between esds in cell parameters are only used when they are defined by crystal symmetry. An approximate (isotropic) treatment of cell esds is used for estimating esds involving l.s. planes.
Refinement. Refinement of F^2^ against ALL reflections. The weighted R-factor wR and goodness of fit S are based on F^2^, conventional R-factors R are based on F, with F set to zero for negative F^2^. The threshold expression of F^2^ > 2sigma(F^2^) is used only for calculating R-factors(gt) etc. and is not relevant to the choice of reflections for refinement. R-factors based on F^2^ are statistically about twice as large as those based on F, and R- factors based on ALL data will be even larger.

### Fractional atomic coordinates and isotropic or equivalent isotropic displacement parameters (Å^2^)

**Table:**

	*x*	*y*	*z*	*U*_iso_*/*U*_eq_	
C1	−0.96779 (13)	−0.27374 (12)	−0.3726 (3)	0.0316 (4)	
C2	−0.93192 (16)	−0.34870 (14)	−0.2418 (3)	0.0415 (4)	
H2	−0.9468	−0.4039	−0.3115	0.050*	
C3	−0.87311 (17)	−0.34058 (17)	−0.0041 (4)	0.0481 (5)	
H3	−0.8474	−0.3903	0.0877	0.058*	
C4	−0.85328 (16)	−0.25859 (17)	0.0941 (4)	0.0469 (5)	
H4	−0.8151	−0.2520	0.2536	0.056*	
C5	−0.89063 (16)	−0.18601 (16)	−0.0462 (3)	0.0429 (5)	
H5	−0.8767	−0.1303	0.0207	0.052*	
N1	−0.94650 (12)	−0.19315 (11)	−0.2767 (2)	0.0331 (3)	
O1	−0.95484 (16)	0.0000	−0.2803 (3)	0.0416 (5)	
O2	−0.75740 (19)	0.0000	−0.7997 (4)	0.0547 (6)	
O3	−0.79963 (14)	−0.07747 (11)	−0.5234 (3)	0.0566 (4)	
O4	−0.63781 (17)	0.0000	−0.4095 (4)	0.0584 (6)	
Cl1	−0.74892 (5)	0.0000	−0.56343 (11)	0.03846 (17)	
Cu1	−1.0000	−0.09408 (2)	−0.5000	0.03551 (13)	
H1	−0.8987 (16)	0.0000	−0.167 (4)	0.053*	

### Atomic displacement parameters (Å^2^)

**Table:**

	*U*^11^	*U*^22^	*U*^33^	*U*^12^	*U*^13^	*U*^23^
C1	0.0339 (8)	0.0310 (9)	0.0312 (9)	−0.0004 (7)	0.0143 (7)	0.0011 (7)
C2	0.0480 (11)	0.0352 (10)	0.0402 (10)	0.0028 (8)	0.0163 (9)	0.0054 (8)
C3	0.0491 (11)	0.0519 (14)	0.0398 (11)	0.0063 (10)	0.0139 (9)	0.0167 (10)
C4	0.0411 (10)	0.0649 (15)	0.0299 (9)	−0.0013 (10)	0.0093 (8)	0.0043 (10)
C5	0.0442 (10)	0.0481 (12)	0.0330 (9)	−0.0068 (9)	0.0118 (8)	−0.0047 (9)
N1	0.0373 (7)	0.0311 (8)	0.0297 (7)	−0.0038 (6)	0.0122 (6)	−0.0013 (6)
O1	0.0478 (11)	0.0305 (10)	0.0335 (10)	0.000	0.0027 (8)	0.000
O2	0.0678 (14)	0.0552 (14)	0.0335 (11)	0.000	0.0123 (10)	0.000
O3	0.0610 (10)	0.0435 (9)	0.0628 (11)	−0.0040 (7)	0.0223 (8)	0.0070 (8)
O4	0.0420 (11)	0.0687 (16)	0.0470 (13)	0.000	−0.0004 (10)	0.000
Cl1	0.0394 (3)	0.0367 (3)	0.0315 (3)	0.000	0.0061 (3)	0.000
Cu1	0.0431 (2)	0.02606 (18)	0.0349 (2)	0.000	0.01302 (14)	0.000

### Geometric parameters (Å, º)

**Table:**

C1—N1	1.345 (2)	N1—Cu1	1.9865 (16)
C1—C2	1.375 (3)	O1—Cu1	1.9097 (13)
C1—C1^i^	1.484 (3)	O1—Cu1^ii^	1.9097 (13)
C2—C3	1.388 (3)	O1—H1	0.807 (10)
C2—H2	0.9300	O2—Cl1	1.440 (2)
C3—C4	1.369 (4)	O3—Cl1	1.4372 (17)
C3—H3	0.9300	O4—Cl1	1.431 (2)
C4—C5	1.376 (3)	Cl1—O3^iii^	1.4372 (17)
C4—H4	0.9300	Cu1—O1^ii^	1.9097 (13)
C5—N1	1.342 (2)	Cu1—N1^i^	1.9865 (16)
C5—H5	0.9300	Cu1—Cu1^ii^	2.8614 (7)
			
N1—C1—C2	121.81 (16)	Cu1—O1—H1	123.4 (12)
N1—C1—C1^i^	114.25 (10)	Cu1^ii^—O1—H1	123.4 (12)
C2—C1—C1^i^	123.94 (11)	O4—Cl1—O3	109.45 (9)
C1—C2—C3	118.8 (2)	O4—Cl1—O3^iii^	109.45 (9)
C1—C2—H2	120.6	O3—Cl1—O3^iii^	110.11 (15)
C3—C2—H2	120.6	O4—Cl1—O2	108.83 (15)
C4—C3—C2	119.3 (2)	O3—Cl1—O2	109.49 (9)
C4—C3—H3	120.3	O3^iii^—Cl1—O2	109.49 (9)
C2—C3—H3	120.3	O1—Cu1—O1^ii^	82.97 (9)
C3—C4—C5	119.13 (19)	O1—Cu1—N1^i^	176.89 (8)
C3—C4—H4	120.4	O1^ii^—Cu1—N1^i^	97.91 (6)
C5—C4—H4	120.4	O1—Cu1—N1	97.91 (6)
N1—C5—C4	122.0 (2)	O1^ii^—Cu1—N1	176.89 (7)
N1—C5—H5	119.0	N1^i^—Cu1—N1	81.37 (9)
C4—C5—H5	119.0	O1—Cu1—Cu1^ii^	41.48 (4)
C5—N1—C1	118.93 (17)	O1^ii^—Cu1—Cu1^ii^	41.48 (4)
C5—N1—Cu1	126.04 (15)	N1^i^—Cu1—Cu1^ii^	139.32 (4)
C1—N1—Cu1	115.01 (11)	N1—Cu1—Cu1^ii^	139.32 (4)
Cu1—O1—Cu1^ii^	97.03 (9)		
			
N1—C1—C2—C3	0.7 (3)	C1^i^—C1—N1—Cu1	−2.8 (2)
C1^i^—C1—C2—C3	−179.4 (2)	Cu1^ii^—O1—Cu1—O1^ii^	0.0
C1—C2—C3—C4	0.6 (3)	Cu1^ii^—O1—Cu1—N1	176.99 (7)
C2—C3—C4—C5	−1.1 (3)	C5—N1—Cu1—O1	−3.53 (17)
C3—C4—C5—N1	0.4 (3)	C1—N1—Cu1—O1	178.01 (13)
C4—C5—N1—C1	0.9 (3)	C5—N1—Cu1—N1^i^	179.53 (19)
C4—C5—N1—Cu1	−177.48 (15)	C1—N1—Cu1—N1^i^	1.07 (9)
C2—C1—N1—C5	−1.4 (3)	C5—N1—Cu1—Cu1^ii^	−0.47 (19)
C1^i^—C1—N1—C5	178.60 (18)	C1—N1—Cu1—Cu1^ii^	−178.93 (9)
C2—C1—N1—Cu1	177.14 (14)		

Symmetry codes: (i) −*x*−2, *y*, −*z*−1; (ii) −*x*−2, −*y*, −*z*−1; (iii) *x*, −*y*, *z*.

### Hydrogen-bond geometry (Å, º)

**Table:**

*D*—H···*A*	*D*—H	H···*A*	*D*···*A*	*D*—H···*A*
O1—H1···O2^iv^	0.81 (2)	2.34 (1)	3.134 (3)	169 (4)
C5—H5···O2^iv^	0.93	2.52	3.381 (3)	153

Symmetry code: (iv) *x*, *y*, *z*+1.
